# Mosquito surveillance for West Nile virus in Connecticut, 2000: isolation from Culex pipiens, Cx. restuans, Cx. salinarius, and Culiseta melanura.

**DOI:** 10.3201/eid0704.010413

**Published:** 2001

**Authors:** T G Andreadis, J F Anderson, C R Vossbrinck

**Affiliations:** Connecticut Agricultural Experiment Station, 123 Huntington Street, New Haven, CT 06504, USA. theodore.andreadis@po.state.ct.us

## Abstract

Fourteen isolations of West Nile (WN) virus were obtained from four mosquito species (Culex pipiens [5], Cx. restuans [4], Cx. salinarius [2], and Culiseta melanura [3]) in statewide surveillance conducted from June through October 2000. Most isolates were obtained from mosquitoes collected in densely populated residential locales in Fairfield and New Haven counties, where the highest rates of dead crow sightings were reported and where WN virus was detected in 1999. Minimum field infection rates per 1,000 mosquitoes ranged from 0.5 to 1.8 (county based) and from 1.3 to 76.9 (site specific). Cx. restuans appears to be important in initiating WN virus transmission among birds in early summer; Cx. pipiens appears to play a greater role in amplifying virus later in the season. Cs. melanura could be important in the circulation of WN virus among birds in sylvan environments; Cx. salinarius is a suspected vector of WN virus to humans and horses.


### Mosquito Surveillance for West Nile Virus in Connecticut, 2000: Isolation from *Culex pipiens, Cx. restuans, Cx. salinarius,* and *Culiseta melanura*

**Theodore G. Andreadis, John F. Anderson, and Charles R. Vossbrinck**
Connecticut Agricultural Experiment Station, New Haven, Connecticut, USA

Fourteen isolations of West Nile (WN) virus were obtained from four mosquito species (*Culex pipiens* [5], *Cx. restuans* [4], *Cx. salinarius* [2], and *Culiseta melanura* [3]) in statewide surveillance conducted from June through October 2000. Most isolates were obtained from mosquitoes collected in densely populated residential locales in Fairfield and New Haven counties, where the highest rates of dead crow sightings were reported and where WN virus was detected in 1999. Minimum field infection rates per 1,000 mosquitoes ranged from 0.5 to 1.8 (county based) and from 1.3 to 76.9 (site specific). *Cx. restuans* appears to be important in initiating WN virus transmission among birds in early summer; *Cx. pipiens* appears to play a greater role in amplifying virus later in the season. *Cs. melanura* could be important in the circulation of WN virus among birds in sylvan environments; *Cx. salinarius* is a suspected vector of WN virus to humans and horses.

Epizootic West Nile (WN) virus activity was first detected in Connecticut during September and October 1999 (1). Substantial die-offs among American Crows, *Corvus brachyrhynchos,* was observed along a 100-km corridor bordering New York State and Long Island Sound in the southwestern corner of the state (lower Fairfield and New Haven counties). During that period, WN virus was isolated from 72 of 86 crows; a Cooper's Hawk, *Accipiter cooperii;* and a Sandhill Crane, *Grus canadensis,* housed at a local zoo (1,2). Expanded mosquito surveillance in the affected region yielded the first isolates of the virus from two species of mosquitoes, *Aedes vexans* and *Culex pipiens* (one pool each), that were trapped in Greenwich, adjacent to the New York border, in mid-September. Despite substantial crow deaths, no additional virus isolates were obtained from >3,500 mosquitoes collected from several hundred traps placed in urban and suburban locations where WN virus-infected crows were found. Neither was WN virus detected in >45,000 mosquitoes (30 species) trapped from June through October in other areas of the state and tested for arboviruses as part of our annual mosquito surveillance program (3). No human or equine cases of WN virus were reported in the state.

In response to these findings, a comprehensive interagency WN virus surveillance and response plan was developed by the state of Connecticut for 2000. The objectives of this program were to detect WN virus, determine the extent of its geographic distribution, and assess the threat to humans and domestic animals. The plan included surveillance for WN virus in mosquitoes, wild birds, domestic animals, poultry, and humans. Mosquito surveillance was specifically designed to identify potential mosquito vectors, determine their seasonal abundance and spatial distribution in the affected area, and assess viral infection rates relative to virus activity in avian and mammalian hosts. The results of this investigation are reported here.

Address for correspondence: Dr. Theodore G. Andreadis, Connecticut Agricultural Experiment Station, 123 Huntington Street, P. O. Box 1106, New Haven, CT 06504 USA; fax: 203-974-8502; e-mail: theodore.andreadis@po.state.ct.us

### Methods

### Mosquito Trapping and Identification

Mosquito trapping was conducted from June 1 through October 26, 2000, at 148 (73 permanent and 75 supplemental) locations statewide (Figure 1). The preexisting mosquito

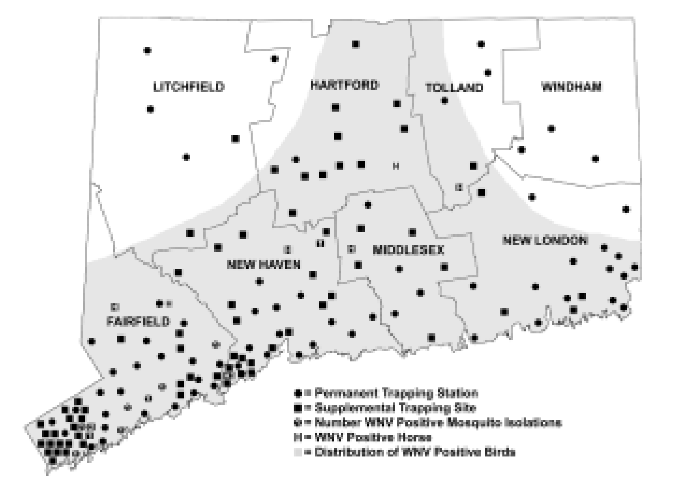

Figure 1. West Nile virus activity in Connecticut, 2000. Locations of mosquito traps, virus isolates from mosquitoes, horse cases, and general distribution of WN virus-positive birds are shown. Source of bird and horse data: Connecticut Departments of Public Health and Agriculture.

surveillance program, consisting of 37 permanent trapping stations principally designed to monitor Eastern equine encephalitis activity (3), was expanded to include 36 new locations, for a total of 73 permanent trap sites. New sites were located in lower Fairfield and New Haven counties, where mosquitoes and dead crows infected with WN virus were found in 1999, and where it was thought that WN virus was most likely to reemerge in 2000. Traps were placed in urban and suburban environs where typical *Culex* spp. habitat was found, including waterways, parks, golf courses, undeveloped wood lots, and temporary wetlands in densely populated residential areas. The 36 preexisting trapping stations in the other six counties (Hartford, Litchfield, Middlesex, New London, Tolland, and Windham) were located mostly in more sparsely populated rural settings that included permanent freshwater swamps (red maple/white cedar), coastal salt marshes, and swamp-forest border locations. Collections were made at 10-day intervals for the entire season (June 1-October 26) at each permanent trap site. The number of trap nights ranged from 12 to 36 (mean 21.7).

Supplemental trapping was conducted at 75 additional locations where dead birds (mostly crows) and horses infected with WN virus were detected during the season and no trapping station was present (Figure 1). These traps were generally placed in the immediate vicinity where the dead birds were recovered in the field or, in the case of the horses, where the animals were stabled. Trapping frequency at the supplemental sites varied; the number of trap nights ranged from 1 to 32 (mean 4.6).

Two trap types were used: 1) a $CO_2$ (dry ice)-baited Centers for Disease Control and Prevention (CDC) light trap and 2) a sod grass-infused CDC gravid mosquito trap (4,5). Typically, traps were placed in the field during the late afternoon and retrieved the following morning. Adult mosquitoes were transported alive to the laboratory, where they were promptly examined on chill tables with a stereo microscope and identified by using descriptions and keys of Darsie and Ward (6) and Means (7,8). Mosquitoes were pooled by species, collecting site, and date. The number of mosquitoes per pool ranged from 1 to 50. In some instances when both trap types were used at the same site on the same evening, mosquito collections were combined. Mosquitoes were stored at -80°C until tested for virus.

### Virus Isolation and Identification

Each frozen mosquito pool was triturated with glass beads and Alundum in 1 mL to 1.5 mL of phosphate-buffered saline containing 0.5% gelatin, 30% rabbit serum, antibiotic, and antimycotic. Following centrifugation for 10 min at 520 x *g*, 100-µL aliquots of each pool of mosquitoes were inoculated onto a monolayer of Vero cells growing in 25-$cm^2$ flasks at 37°C in 5% $CO_2$. Cells were examined for cytopathologic effect for up to 7 days after inoculation. Uninoculated flasks were kept as negative controls.

Virus isolates were identified by enzyme immunoassay (ELISA), reverse transcription-polymerase chain reaction (RT-PCR), or both. Reference antibodies for the ELISA were prepared in mice (9) and provided by the World Health Organization Center for Arbovirus Research and Reference, Yale Arbovirus Research Unit, Department of Epidemiology and Public Health, Yale University School of Medicine. These included seven viruses, in three families, isolated from mosquitoes in North America: Cache Valley, Eastern equine encephalitis, Highlands J, Jamestown Canyon, La Crosse, St. Louis encephalitis, and WN virus. Positive and negative control cell lysates were included in each test.

For molecular identification, Vero cell cultures showing lytic activity were pelleted and processed by using a Qiagen Rneasy mini protocol. The Rneasy column was eluted twice with 40 µL of RNase-free cell culture water. Two microliters of the column eluate was reverse transcriptase amplified by using the Perkin-Elmer GeneAmp EZ rTh RNA PCR kit (Norwalk, CN). Three sets of primers representing five primer sites unique to WN virus were used for redundancy: 1) WN-233F (GACTGAAGAGGGCAATGTTGAGC) and WN-1189R (GCAATAACTGCGGACYTCTGC); 2) WN-200F (TCAATATGCTAAAACGCGG) and WN-540R (TTAGAGAGGGTAACTGCTCC); and 3) WN-451F (GTGCTATCAATCGGCGGAGCTC) and 540R. Gene amplification was done on an MJ Research PTC-200 DNA Engine (Waltham, MA). The protocol was as follows: 60°C for 30 min, 94°C for 2 min followed by 40 cycles of 94°C for 45 sec, 50°C for 30 sec, and 60°C for 1 min 30 sec. PCR product was run in a 1.5% agarose gel stained with ethidium bromide and electrophoresed at 20 V/CM for approximately 1/2 hr. Band size was checked against the AmpliSize size markers from Bio-Rad Laboratories (Richmond, CA). All WN virus isolates were confirmed by RT-PCR.

### Results

Mosquito collection data are summarized in Table 1. A total of 137,199 female mosquitoes representing 32 species in

Table 1. Total number of mosquito species trapped and tested for West Nile virus in Connecticut, June 1–October 26, 2000

| Mosquito species | No. locations | No. collected and tested | No. pools |
|---|---|---|---|
| *Aedes cinereus* | 104 | 9,195 | 641 |
| *Ae. vexans* | 125 | 8,310 | 622 |
| *Anopheles barberi* | 4 | 5 | 5 |
| *An. crucians* | 1 | 6 | 1 |
| *An. punctipennis* | 126 | 2,477 | 516 |
| *An. quadrimaculatus* | 35 | 98 | 53 |
| *An. walkeri* | 31 | 380 | 82 |
| *Coquillettidia perturbans* | 95 | 11,516 | 536 |
| *Culex pipiens* | 125 | 4,399 | 473 |
| *Cx. restuans* | 84 | 4,690 | 468 |
| *Cx. salinarius* | 100 | 6,673 | 466 |
| *Cx. territans* | 26 | 46 | 36 |
| *Culiseta melanura* | 108 | 8,105 | 625 |
| *Cs. morsitans* | 39 | 271 | 79 |
| *Ochlerotatus abserratus* | 57 | 1,605 | 136 |
| *Oc. atropalpus* | 1 | 1 | 1 |
| *Oc. aurifer* | 56 | 3,164 | 187 |
| *Oc. canadensis* | 101 | 29,172 | 1,141 |
| *Oc. cantator* | 79 | 3,514 | 322 |
| *Oc. communis* | 5 | 127 | 8 |
| *Oc. excrucians* | 59 | 921 | 146 |
| *Oc. grossbecki* | 1 | 1 | 1 |
| *Oc. japonicus* | 82 | 690 | 250 |
| *Oc. sollicitans* | 21 | 1,855 | 90 |
| *Oc. sticticus* | 63 | 9,054 | 327 |
| *Oc. stimulans* | 30 | 257 | 51 |
| *Oc. taeniorhynchus* | 13 | 5,978 | 153 |
| *Oc. triseriatus* | 113 | 1,711 | 418 |
| *Oc. trivittatus* | 119 | 19,260 | 761 |
| *Orthopodomyia signifera* | 5 | 5 | 5 |
| *Psorophora ferox* | 82 | 2,361 | 233 |
| *Uranotaenia sapphirina* | 99 | 1,352 | 252 |
| Totals | | 137,199 | 9,085 |

eight genera were collected from the field, identified, and processed for virus isolation. Fifteen species of *Ochlerotatus* (formerly *Aedes*) and two species of *Aedes* were collected, among which *Ochlerotatus canadensis* and *Oc. trivittatus* were the most abundant, followed by *Aedes cinereus, Oc. sticticus, Ae. vexans,* and *Oc. taeniorhychus.* With the exception of *Oc. taeniorhychus* (a salt marsh inhabitant) and to a lesser degree *Oc. sticticus,* each of these species was widely distributed. Of four species of *Culex* collected, *Cx. salinarius* was the most numerous. *Cx. pipiens* and *Culex restuans* were less abundant but were equal in number. Other notably abundant species included *Coquillettidia perturbans, Culiseta melanura, Anopheles punctipennis,* and *Psorophora ferox.*

Virus isolation data are summarized (Table 2, Figure 1). Fourteen isolates of WN virus were obtained from four mosquito species: *Cx. pipiens* (5 isolates), *Cx. restuans* (4 isolates), *Cx. salinarius* (2 isolates), and *Cs. melanura* (3 isolates). Infected mosquitoes were recovered from 11 locations. With the exception of the positive pool from Meriden, a town in northern New Haven County, all isolates were obtained from mosquitoes collected from lower Fairfield and New Haven counties in the southwestern corner of the state, bordering Long Island Sound. The first isolate was obtained from *Cx. restuans* collected on July 11 and the last from *Cs. melanura* collected on October 2. Most (9 of 14) of the isolations were made from mosquitoes collected in mid-September. Minimum field infection rates calculated from season-long collections in each county ranged from 1.8 per 1,000 for *Cx. restuans* to 0.5 for *Cx. salinarius.* Site-specific minimum field infection rates ranged from 1.3 to 76.9. *Culex* spp. infected with WN virus were collected in traps set in densely populated suburban areas (mean population density 2,431 people/sq. mile). *Cs. melanura* infected with WN virus, by contrast, were collected from semipermanent swamp habitats in less populated locales (mean population density 1,407 people/sq. mile). Seven of the 11 locations where infected mosquitoes were found on one occasion only during the season were permanent trapping stations that were monitored from June through October. The number of trap nights at these sites ranged from 26 to 36 (mean 28.6). The trapping effort at the four supplemental sites where isolations were made ranged from 10 to 32 trap nights (mean 15.0).

Isolations from multiple pools of mosquitoes collected at the same site were obtained at Milford and Stamford-2 (Table 2). The Milford site (three isolates) was a stable in a densely populated industrial area adjacent to an isolated wood lot where a horse was diagnosed with WN virus (onset September 4). The first isolate was from a pool of *Cx. salinarius* collected on September 18. Two additional isolates were obtained from *Cx. pipiens* and *Cx. salinarius* collected on September 21. No further isolations were made from mosquitoes collected in traps set at this location on September 27 and October 4. The Stamford-2 site was a small wood lot in a densely populated area. Trapping was conducted on September 13, 20, and 27 and October 3 and 24. Two isolations were obtained from *Cx. pipiens* and *Cx. restuans* collected on September 20.

The weekly collection data for those mosquitoes from which WN virus was isolated (*Cx. restuans, Cx. pipiens, Cx. salinarius* and *Cs. melanura*) are shown (Figure 2). *Cx. restuans* was notably more abundant during early summer (June and July, peak in early July) and was rarely collected in August and September. *Cx. pipiens*, on the other hand, was present in July but was clearly more abundant later in the summer (August and September, peak in late August). With the exception of the early WN virus isolation from *Cx. restuans* in mid-July, all viruses from these two species were isolated when populations of both mosquitoes were on the decline.

*Cx. salinarius* populations peaked in mid-July and steadily but gradually declined through October. *Cs. melanura* was consistently collected throughout the entire season but there were two discernible peaks of adult abundance, early June and mid-August. WN virus was isolated from both species on the same week in mid-September, when populations were similarly declining.

### Conclusion

Our isolations of WN virus from mosquitoes collected in coastal Fairfield and New Haven counties were consistent with epizootic WN virus activity in this region during 2000. Although wild birds (mostly crows) infected with WN virus were recovered throughout south-central Connecticut, the highest rates of dead crow sightings reported (10) were consistently noted in those areas where 13 of 14 mosquito isolations were made. This was also the same general area where WN virus was initially detected in American crows and mosquitoes in 1999 (1). These findings, in concert with the limited flight range of crows during the early summer (11) and

Table 2. West Nile virus isolation data from field-collected mosquitoes trapped in Connecticut, June 1–October 26, 2000

| Species | Date collected | Pool size | Location: County | Location: Site | MFIR[a]: County | MFIR[a]: Site | Trap type[b] |
|---|---|---|---|---|---|---|---|
| *Culex restuans* | 7/11 | 9 | Fairfield | Stamford-1 | 1.8 | 6.9 | G |
| | 8/7 | 3 | | Norwalk-1 | | 32.3 | G,L |
| | 8/7 | 7 | | Norwalk-2 | | 5.4 | G,L |
| | 9/20 | 18 | | Stamford-2 | | 55.6 | G,L |
| *Cx. pipiens* | 8/30 | 1 | Fairfield | Greenwich | 1.3 | 29.4 | G |
| | 9/11 | 44 | | Stamford-3 | | 17.2 | G |
| | 9/20 | 50 | | Stamford-2 | | 15.9 | G,L |
| | 9/12 | 4 | New Haven | Meriden | 1.4 | 41.7 | L |
| | 9/21 | 3 | | Milford | | 76.9 | G,L |
| *Cx. salinarius* | 9/18 | 5 | New Haven | Milford | 0.5 | 45.5 | L |
| | 9/21 | 6 | | Milford | | 45.5 | L |
| *Culiseta melanura* | 9/19 | 39 | Fairfield | Fairfield | 0.8 | 9.2 | L |
| | 9/20 | 50 | | Shelton | | 1.3 | L |
| | 10/2 | 7 | | Westport | | 6.8 | L |

[a]Minimum field infection rate per 1,000 mosquitoes.
[b]G = gravid; L = light; G,L = combined.

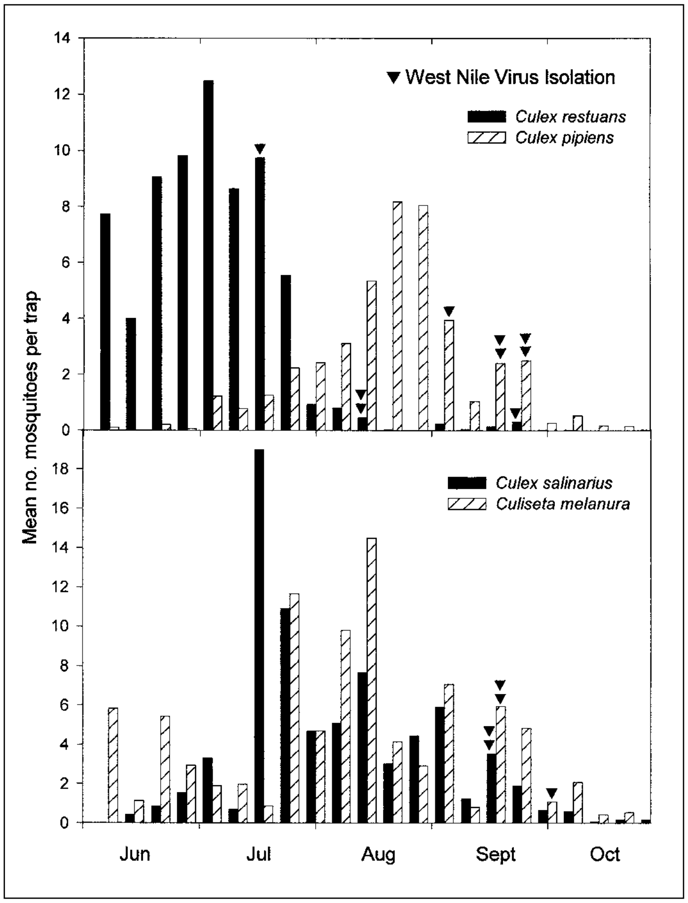

Figure 2. Weekly collection and West Nile virus isolation data for field-collected adult female *Culex restuans, Cx. pipiens, Cx. salinarius,* and *Culiseta melanura* in Connecticut, 2000.

isolation from *Cx. restuans* in mid-July, suggest local reemergence and transmission of the virus in this region, independent of the early seasonal events in New York and New Jersey (12). It is uncertain, however, whether early amplification in this region led to the subsequent spread of the virus to other areas of the state. The mechanism for overwintering of WN virus is also unknown. The detection of WN virus in hibernating *Culex* spp. mosquitoes collected in New York City during January-February (13) and the demonstration of vertical transmission of the virus by mosquitoes in the laboratory (14) and field (15) suggest that vertical transmission could provide a mechanism for persistence of the virus during the winter months.

The relative importance of various mosquitoes as epidemic and epizootic vectors of WN virus in North America is largely unknown. Investigations in Africa, Europe, and Asia (16) have mostly incriminated bird-feeding species, predominantly of the genus *Culex* spp., as the main vectors. Tsai et al. (17) and Savage et al. (18) have suggested that WN virus circulates in Europe in both sylvan and urban transmission cycles involving different species and populations of mosquitoes. In the sylvatic cycle, WN virus is circulated among birds by *Cx. modestus*, *Cx. pipiens*, or both. Because *Cx. modestus* displays a broad host range, it may also transmit the virus to humans. *Cx. pipiens,* on the other hand, is strongly ornithophilic and appears to be more important in amplification of the virus among birds than in transmission to humans in these natural environs. However, in urban areas, *Cx. pipiens* is the only common *Culex* mosquito and is believed to serve both functions.

Our isolates from *Cx. pipiens, Cx. restuans,* and *Cx. salinarius* collected in densely populated communities are consistent with these reports and agree with the preponderance of WN virus-positive pools (406 of 456) obtained from *Culex* species collected from other northeastern states in 2000 (19). The isolations from *Cs. melanura* collected in more rural environs are new host records for WN virus. If proven to be a competent vector, this almost exclusively avian feeder could be important in circulation of the virus among birds in sylvan environments.

The multiple isolates from *Cx. restuans* and *Cx. pipiens* support our hypothesis that these species are important enzootic and epizootic vectors. Both species are strongly ornithophilic (20-25), are widely distributed throughout the region, and occur in both urban and rural environs. Recently completed studies (26,27) have further demonstrated that *Cx. pipiens* is a competent vector for WN virus in the laboratory. The competence of *Cx. restuans* has not been established.

*Cx. restuans* may be important in initiation of WN virus transmission among wild birds in early summer. It is the most abundant *Culex* species in June and July, and the earliest isolates were from this species in July and August. In contrast, *Cx. pipiens* became abundant in August, with isolations made on August 30 and in September. *Cx. pipiens* may therefore play a greater role in amplification of WN virus later in the season. Reiter (28) has suggested that, in the east-central United States, where *Cx. restuans* populations typically peak in mid-May, this species may play a similar role in recrudescence and early amplification of St. Louis encephalitis virus in the spring. He further speculates that reactivation of previously infected female *Cx. restuans* during periods of unseasonably cold weather in the summer, when it normally estivates, could cause a sudden, synchronous release of virus at a time when it could then be amplified by an increasing *Cx. pipiens* population that peaks in early to mid-July.

The role that *Cx. pipiens* and *Cx. restuans* may play in transmission of WN virus to humans, horses, or other mammals is unclear. Most reports (8,20-25) indicate that both species predominately feed on birds and are reluctant to feed on humans. Blood meal analysis of local populations in Connecticut (25) has further shown that *Cx. pipiens* and *Cx. restuans* acquire blood almost exclusively from passeriform birds. Similar results have been reported for *Cx. pipiens* populations in New York (24) and New Jersey (21). On the other hand, several researchers (8,20,22,29,30) have reported that when *Cx. restuans* is abundant, females will bite wild and domestic animals, and humans. We note that WN virus was isolated from two pools of *Cx. restuans* mosquitoes collected from two locations in Norwalk in Fairfield County on August 7 (Table 2). This was the same community where a mildly symptomatic woman was diagnosed with WN virus with onset in late August (10,19).

Differences in host feeding preferences have also been observed in farm and woodland populations of *Cx. pipiens* in the northeastern United States (22). According to Means (22), *Cx. pipiens* inhabiting commercial bird farms routinely engorge on ducks and pheasants but hardly ever bite humans, but populations in sylvan environments attack humans readily. The human biting behavior of the urban *molestus*

form of *Cx. pipiens (*which breeds in basements, subways, and similar dark, heated places [31]) also cannot be discounted. However, we have no knowledge of the identity, abundance, or distribution of this behavioral form of *Cx. pipiens* in Connecticut. Clearly, more research on the host feeding preferences of these two mosquitoes is needed.

*Cx. salinarius,* by contrast, is a well-recognized general feeder that feeds indiscriminately on both birds and mammals and will readily bite humans (8,21,30,32,33). In addition to the two isolates reported here, WN virus was detected in 33 pools of this mosquito collected from other areas of the Northeast in 2000 (19). Our two isolates were from females collected at a stable where a horse was diagnosed with WN virus. *Cx. salinarius* should be strongly considered as a possible vector of WN virus to humans, horses, and other animals.

### Acknowledgments

We acknowledge the technical assistance of John Shepard and Michael Thomas (mosquito collection and identification); Jodie Correia, Bonnie Hamid, and Michael Vasil (virus isolation and serology); and Melanie Baron (reverse transcription-polymerase chain reaction). We also thank the following for assistance in collecting and processing mosquitoes: Daniel Altneu, Dawn Berube, Edward Calandella, John Capotosto, Eric Carlson, John Duarte, Grecekia Elliot, Ronald Ferrucci Jr., Hannah Ginese, Michael MacAloon, Laura Mickowski, Ryan Monroe, Lisa Nigro, Kelly Shanley, Michael Spada, the Stamford Health Department, Ledgelight Health District, U.S. Navy, and Integrated Mosquito Control.

This work was supported in part by Epidemiology and Laboratory Capacity for Infectious Diseases cooperative agreement number U50/CCU116806-01-1 from the Centers for Disease Control and Prevention.

Dr. Andreadis is chief medical entomologist at the Connecticut Agricultural Experiment Station in New Haven, Connecticut. His research interests include epidemiology of vector-borne diseases, mosquito ecology, insect pathology, and microbial control of mosquitoes.

### References

1. Anderson JF, Andreadis TG, Vossbrinck CR, Tirrell S, Wakem EM, French RA, et al. Isolation of West Nile virus from mosquitoes, crows, and a Cooper's Hawk in Connecticut. Science 1999;286:2331-3.
2. Lanciotti RS, Roehrig JT, Deubel V, Smith J, Parker M, Steele K, et al. Origin of the West Nile virus responsible for an outbreak of encephalitis in the northeastern United States. Science 1999;286;2333-7.
3. Andreadis TG, Anderson JF, Vossbrinck CR. Mosquito arbovirus surveillance in Connecticut, 1999: Isolation and identification of West Nile virus. Proceedings Northeastern Mosquito Control Association 1999;45:57-67.
4. Lampman RL, Novak RJ. Oviposition preferences of *Culex pipiens* and *Culex restuans* for infusion-baited traps. J Am Mosq Control Assoc 1996;12:23-32.
5. Reiter P. A portable, battery-powered trap for collecting gravid *Culex* mosquitoes. Mosquito News 1983;43:496-8.
6. Darsie RF Jr, Ward RA. Identification and geographic distribution of mosquitoes of North America, north of Mexico. Mosquito Systematics Supplement, American Mosquito Control Association 1981;1:1-313.
7. Means RG. Mosquitoes of New York. Part I. The genus *Aedes* Meigen with identification keys to genera of Culicidae. New York State Museum Bulletin 1979;430a.
8. Means RG. Mosquitoes of New York. Part II. Genera of Culicidae other than *Aedes* occurring in New York. New York State Museum Bulletin 1987;430b.
9. Ansari MZ, Shope RE, Malik S. Evaluation of Vero cell lysate antigen for ELISA of flaviviruses. J Clin Lab Anal 1993;7:230-7.
10. Hadler J, Nelson R, McCarthy T, Andreadis T, Lis MJ, French R, et al. West Nile virus surveillance in Connecticut in 2000: An intense epizootic without high risk for severe human disease. Emerg Infect Dis 2001;7:636-42.
11. Caceamise DF, Reed LM, Romanowski J, Stauffer PC. Roosting behavior and group territoriality in American crows. The Auk 1997;114:628-37.
12. Centers for Disease Control and Prevention. Update: West Nile virus activity--New York and New Jersey, 2000. MMWR Morb Mortal Wkly Rep 2000;49:640-2.
13. Centers for Disease Control and Prevention. Update: surveillance for West Nile virus in overwintering mosquitoes--New York, 2000. MMWR Morb Mortal Wkly Rep 2000;49:178-9.
14. Baqar S, Hayes CG, Murphy JR, Watts DM. Vertical transmission of West Nile virus by *Culex* and *Aedes* species mosquitoes. Am J Trop Med Hyg 1993;48:757-62.
15. Miller BR, Nasci RS, Godsey MS, Savage HM, Lutwama JJ, Lanciotti RS, et al. First field evidence for natural vertical transmission of West Nile virus in *Culex univittatus* complex mosquitoes from Rift Valley Province, Kenya. Am J Trop Med Hyg 2000;62:240-6.
16. Hubalek Z, Halouzka J. West Nile fever-a reemerging mosquito-borne viral disease in Europe. Emerg Infect Dis 1999;5:643-50.
17. Tsai TF, Popovici F, Cernescu C, Campbell GL, Nedelcu NI. West Nile encephalitis epidemic in southeastern Romania. Lancet 1998;352:767-71.
18. Savage HM, Ceianu C, Nicolescu G, Karabatsos N, Lanciotti R, Vladimirescu A, et al. Entomologic and avian investigations of an epidemic of West Nile fever in Romania in 1996, with serologic and molecular characterization of a virus isolate from mosquitoes. Am J Trop Med Hyg 1999;61:600-11.
19. Centers for Disease Control and Prevention. Update: West Nile virus activity—eastern United States, 2000. MMWR Morb Mortal Wkly Rep 2000;49:1044-7.
20. Hayes RO. Host preferences of *Culiseta melanura* and allied mosquitoes. Mosquito News 1961;179-87.
21. Crans WJ. Continued host preference studies with New Jersey mosquitoes, 1963. In: Proceedings of the 51st Annual Meeting of the New Jersey Mosquito Extermination Association; 1963. p. 50-8.
22. Means RG. Host preferences of mosquitoes (Diptera: Culicidae) in Suffolk County, New York. Ann Entomol Soc Am 1968;61:116-20.
23. Edman JD. Host-feeding patterns of Florida mosquitoes III. *Culex (Culex)* and *Culex (Neoculex)*. J Med Entomol 11;1975:635-53.
24. Tempalis CA. Host-feeding patterns of mosquitoes, with a review of advances in analysis of blood meals by serology. J Med Entomol 11;1975:635-53.
25. Magnarelli LA. Host feeding patterns of Connecticut mosquitoes. Am J Trop Med Hyg 1977;26:547-52.
26. Turell MJ, O'Guinn ML, Oliver J. Potential for New York mosquitoes to transmit West Nile virus. Am J Trop Med Hyg 2000;62:413-4.
27. Turell MJ, O'Guinn ML, Dohm B, Jones JW. Vector competence of North American mosquitoes (Diptera: Culicidae) for West Nile virus. J Med Entomol 2001;38:130-4
28. Reiter P. Weather, vector biology, and arboviral recrudescence. In: Monath TP, editor. Arboviruses: epidemiology and ecology. Vol 1. Boca Raton: CRC Press; 1988. p. 245-55.
29. Barr AR. The mosquitoes of Minnesota. University of Minnesota Agricultural Experiment Station Technical Bulletin 1958;228.
30. Murphey FJ, Burbutis PP, Bray DF. Bionomics of *Culex salinarius* Coquillett. II. Host acceptance and feeding by adult females of *C. salinarius* and other mosquito species. Mosquito News 1967;27:366-74.
31. Burbutis PP, Jobbins DM. Notes on certain characteristics of two populations of *Culex pipiens* Linn. In: Proceedings of the 50th Annual Meeting of the New Jersey Mosquito Extermination Association; 1963. p. 289-97.
32. Edman JD. Host-feeding patterns of Florida mosquitoes III. *Culex (Culex)* and *Culex (Neoculex)*. J Med Entomol 1974;11:95-104.
33. Cupp EW, Stokes GM. Feeding patterns of *Culex salinarius* Coquillett in Jefferson Parish, Louisiana. Mosquito News 1976;36:332-5.
